# Controlled Forgetting: Targeted Stimulation and Dopaminergic Plasticity Modulation for Unsupervised Lifelong Learning in Spiking Neural Networks

**DOI:** 10.3389/fnins.2020.00007

**Published:** 2020-01-28

**Authors:** Jason M. Allred, Kaushik Roy

**Affiliations:** Nanoelectronics Research Laboratory, Electrical and Computer Engineering Department, Purdue University, West Lafayette, IN, United States

**Keywords:** lifelong learning, continual learning, catastrophic forgetting, controlled forgetting, dopaminergic learning, Spiking Neural Networks, Spike Timing Dependent Plasticity, stability-plasticity dilemma

## Abstract

Stochastic gradient descent requires that training samples be drawn from a uniformly random distribution of the data. For a deployed system that must learn online from an uncontrolled and unknown environment, the ordering of input samples often fails to meet this criterion, making lifelong learning a difficult challenge. We exploit the locality of the unsupervised Spike Timing Dependent Plasticity (STDP) learning rule to target local representations in a Spiking Neural Network (SNN) to adapt to novel information while protecting essential information in the remainder of the SNN from catastrophic forgetting. In our Controlled Forgetting Networks (CFNs), novel information triggers stimulated firing and heterogeneously modulated plasticity, inspired by biological dopamine signals, to cause rapid and isolated adaptation in the synapses of neurons associated with outlier information. This targeting controls the forgetting process in a way that reduces the degradation of accuracy for older tasks while learning new tasks. Our experimental results on the MNIST dataset validate the capability of CFNs to learn successfully over time from an unknown, changing environment, achieving 95.24% accuracy, which we believe is the best unsupervised accuracy ever achieved by a fixed-size, single-layer SNN on a completely disjoint MNIST dataset.

## 1. Introduction

Artificial neural networks have enabled computing systems to successfully perform tasks previously out of reach for traditional computing, such as image and audio classification. These networks, however, are typically trained offline and do not update during deployed inference. One of the current obstacles preventing fully autonomous, unsupervised learning in dynamic environments while maintaining efficiency is the *stability-plasticity dilemma*, or the challenge of ensuring that the system can continue to quickly and successfully learn from and adapt to its current environment while simultaneously retaining and applying essential knowledge from previous environments (Grossberg, 1987).

There have been a handful of terms used in literature to describe the process of learning from data that is temporally distributed inhomogeneously, such as the terms incremental learning, sequential learning, continual learning, and lifelong learning. In this work, we will use the term “lifelong learning.” *Lifelong learning* is the process of successfully learning from new data while retaining useful knowledge from previously encountered data that is statistically different, often with the goal of sequentially learning differing tasks while retaining the capability to perform previously learned tasks without requiring retraining on data for older tasks. When traditional artificial neural networks are presented with changing data distributions, more rigid parameters interfere with adaption, while more flexibility causes the system to fail to retain important older information, a problem called *catastrophic interference* or *catastrophic forgetting*. Biological neuronal systems dont seem to suffer from this dilemma. We take inspiration from the brain to help overcome this obstacle.

To avoid catastrophic forgetting, important information from older data must be protected while new information is learned from novel data. Non-local learning rules may not provide such isolation. Localized learning, on the other hand, may provide the desired segmentation while also being able to perform unsupervised learning, which is critical for lifelong learning in unknown environments. Spike Timing Dependent Plasticity (STDP) is a localized biological Hebbian learning process where a synaptic weight's adjustment is a function of the timing of the *spikes*, or firing events, of its locally connected pre- and post-synaptic neurons. Spiking Neural Networks (SNNs), which have been explored for their potential energy advantages due to sparse computing (Han et al., 2018), have been shown to perform successful unsupervised clustering tasks with STDP (Diehl and Cook, 2015).

However, even though STDP learning is localized, it is still susceptible to catastrophic forgetting because the algorithms that employ STDP are traditionally designed for randomized input ordering. Certain features, such as homeostasis, attempt to distribute the effect of input groupings globally in order to benefit from the full network. Without a temporally uniform distribution of classes, traditional STDP algorithms still lose important older information, which is either replaced by or corrupted with information from newer samples (Allred and Roy, 2016).

We present a new learning paradigm, inspired by the dopamine signals in mammalian brains that non-uniformly, or heterogeneously modulate synaptic plasticity. We create Controlled Forgetting Networks (CFNs) that address the stability-plasticity dilemma with rapid/local learning from new information, rather than the traditional gradual/global approach to learning. Our approach allows fixed-size CFNs to successfully perform unsupervised learning of sequentially presented tasks without catastrophically forgetting older tasks.

Many recent papers have tackled the challenge of lifelong learning without catastrophic forgetting, but they are not designed to target the goal of this paper, which is autonomous learning on a deployed neuromorphic system. This goal requires real-time unsupervised learning, energy efficiency, and fixed network resources. Wysoski et al. (2006), Srivastava et al. (2013), Wang et al. (2014), Wang et al. (2015), Rusu et al. (2016), Fernando et al. (2017), Kirkpatrick et al. (2017), Lee et al. (2017), Aljundi et al. (2018), Li and Hoiem (2018), Bashivan et al. (2019) and Du et al. (2019) all employ supervised or reinforcement learning methods, in some way provide the network with the knowledge of when a task change occurs, or provide access to previous samples for retraining. For example, the work by Aljundi et al. (2018) requires that the system be allowed a parameter-“importance update” period on the older task(s) before proceeding to a new task. Similarly, Panda et al. (2018) requires that samples from earlier distributions be presented in disproportionately larger quantities than later distributions to avoid catastrophic forgetting, which would require knowledge of a task change. Additionally, Srivastava et al. (2013), Rusu et al. (2016), Fernando et al. (2017), Kirkpatrick et al. (2017), Lee et al. (2017), Li and Hoiem (2018) and Rios and Itti (2018) are also not applicable to localized learning rules that may be employed on spiking networks. And Wysoski et al. (2006), Dhoble et al. (2012), and Wang et al. (2017) are morphological systems that do not work with static-sized networks, which would exclude them from direct mapping onto physical hardware implementations.

## 2. Materials and Methods

### 2.1. The Challenge of Lifelong Learning

Backpropagation has proven a successful learning algorithm for deep neural networks. The accuracy of this approach depends on proper stochastic gradient descent or SGD, also known as incremental gradient descent, in which many small, global adjustments to network weights are performed while iterating over samples from a training dataset. These samples, however, must be drawn from a random distribution of the dataset—hence the name “stochastic” gradient descent—intermixing the classes so that each class can affect the direction of descent for correct error minimization throughout the entire training process.

The need to draw training samples from a random distribution is an obstacle for on-line learning, especially when the system encounters novel data. Backpropagation in an on-line system for real-time learning proves difficult when the input from the environment is uncontrolled and unknown. With traditional SGD, the system typically has three choices to attempt learning from novel data: (1) train normally on inputs in the order seen; (2) periodically go offline and retrain from an updated dataset; (3) maintain an online storage of previous samples to intermix with the new samples, providing a simulated random sampling. The latter two choices are costly and inhibit real-time learning, while the first catastrophically violates SGD.

#### 2.1.1. Catastrophic Forgetting Due to Global Interference

If a uniformly randomized order is not provided, e.g., samples are grouped by class and classes are presented sequentially to the network, then the gradient descent followed by latter samples will likely disagree with the direction from previous samples. This conflict causes the network to fail to reach an error minimum that respects older tasks, as at each period of time in the training process the network essentially attempts to globally optimize for only the current tasks, agnostic as to whether or not that particular direction increases the error for older tasks. Latter samples erase or corrupt the information learned from previous samples, causing catastrophic forgetting.

One of the largest underlying causes of catastrophic forgetting in backpropagation algorithms is the reliance on a global error. Calculating weight updates from the current sample's global error means that the current sample may globally affect network weights. Biological neuronal learning, on the other hand, appears to be significantly localized, with synaptic weight updates being a function of local activity, causing different regions to be responsible for different tasks. While distributed representations promote generalization in neural networks, rapid learning of novel information may not require significant modifications to low-level distributed representations in a sufficiently trained network. It has been shown that the IT cortex contains a large-scale spatial organization, or “shape map,” that remains significantly stable over time (Op de Beeck et al., 2007), even while learning novel information. Lee and DiCarlo (2019) have shown that the stable earlier levels of the visual cortex are capable of representing the generic structure and composition of never-before-seen inputs with an already-learned understanding of the physical world that remains constant through the remainder of life–for example, an understanding of lines, edges, curves, and colors at the lowest levels and an understanding of rotations, shading, and physical properties at subsequent levels. Thus, it is likely that lifelong learning need only occur in the last one or two layers of a neural network, where local learning may sufficiently classify from a read-out of the higher-dimensional generalizations that have been learned previously.

#### 2.1.2. Catastrophic Forgetting in Localized Learning Due to Homeostasis

Many leading STDP-trained SNNs employ adaptive thresholding, in which a neuron's firing threshold increases each time it fires and otherwise decays, preventing specific neurons from dominating the receptive field. Adaptive thresholding helps achieve homeostasis by distributing the firing activity between neurons. However, adaptive thresholding assumes a temporally random distribution of input samples and often causes catastrophic interference when the environment changes (Allred and Roy, 2016). For lifelong learning, adaptive thresholding must be modified to account for long-term variations in spiking activity that would occur when processing temporally variant input distributions.

#### 2.1.3. The Need for Forgetting

For successful lifelong learning, there must be network resources available to learn new information. In a deployed system with finite resources, some forgetting of older knowledge is required to make room for information from new data. As mentioned earlier, there are morphological systems that logically grow the network to accommodate new information, even employing pruning techniques when necessary if the network grows too large. However, for our goal of deployed learning on neuromorphic hardware, inserting and removing physical components of the network is not an option, and existing network components must be re-purposed to learn a new task when network capacity is reached, requiring some forgetting.

Additionally, in some cases, forgetting may actually be beneficial. Forgetting outlier data can improve generalizations, and forgetting stale data can allow the system to adapt to a changing environment if new information directly contradicts older information. Because some forgetting must occur, we seek to control the forgetting process to protect the most vital information, minimizing accuracy loss.

### 2.2. Controlled Forgetting With Dopaminergic Learning

**Figure 1:**
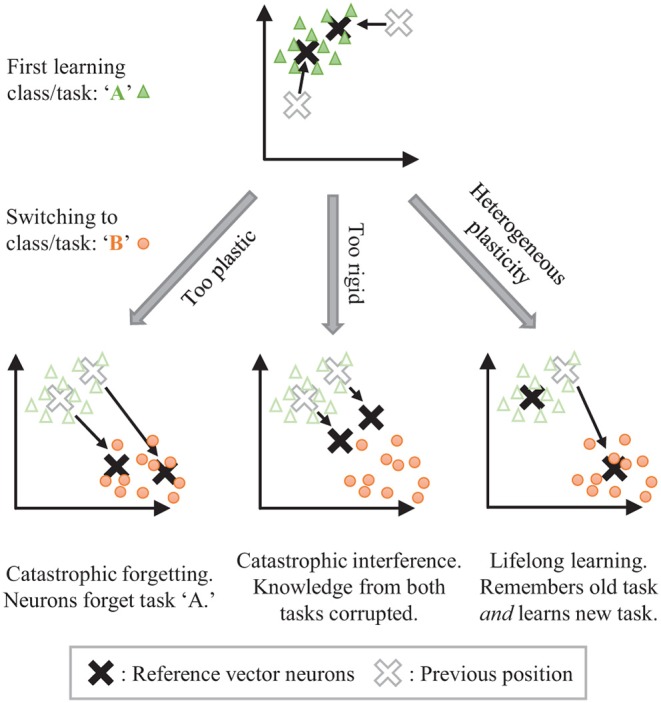
The stability-plasticity dilemma in unsupervised clustering. Lifelong learning is achieved with a strategic heterogeneous modulation of synaptic plasticity.

The stability-plasticity dilemma can be addressed by allowing for dynamic, heterogeneously modulated plasticity. Consider the example of unsupervised clustering where neurons are trained to center on input clusters (see Figure 1). Temporarily making the synaptic weights of some neurons more plastic while keeping the weights of other neurons more rigid can allow for isolated adaptation by the plastic parameters while protecting the information associated with the rigid parameters. The challenge then becomes how to dynamically control the plasticity and for which parameters.

STDP embeds local, generalized representations of correlated inputs within the synaptic weights of individual neurons. Lateral inhibition between neurons, similar to the architecture in Diehl and Cook (2015), creates competition that prevents multiple neurons from learning the same information. We seek to control the forgetting process by harnessing the segmentation of localized and distinct representations that are created by STDP with competition. Interference from novel information may be isolated by stimulating specific network elements to adapt to that information, protecting the remainder of the network from change. The forgetting caused by this interference may be minimized and controlled by targeting network elements associated with less useful information. We draw on inspiration from biology to heterogeneously modulate STDP learning to perform such isolated adaptation, creating Controlled Forgetting Networks (CFNs).

#### 2.2.1. Biologically Inspired Dopaminergic Plasticity Modulation

**Figure 2:**
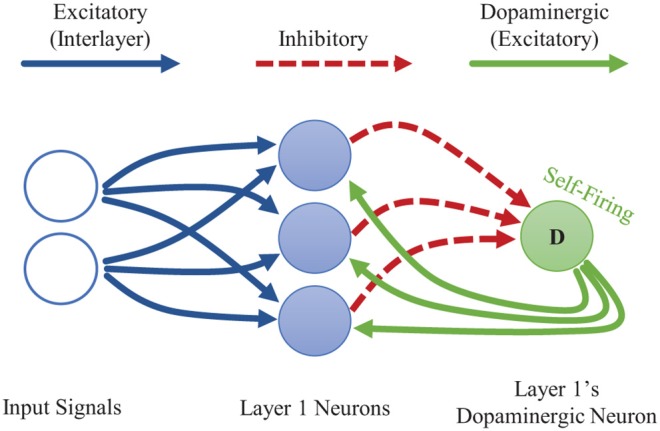
Single-layer CFN architecture. The dopaminergic neuron fires when the other neurons on its layer are *not* firing, often a sign of novel information. The firing of the dopaminergic neuron stimulates firing in the other neurons while temporarily enhancing plasticity. The stimulation signals from the dopaminergic neuron are weighted to provide heterogeneous, targeted stimulation. The other neurons within a layer each have additional laterally inhibitory connections for competition (not shown here).

Dopamine acts as a neuromodulator which gates synaptic plasticity. Dopamine signals are most commonly thought of as reward signals. In addition, though, dopamine releases are also associated with encountering novel data, which allows the brain to quickly adapt to new information (Frémaux and Gerstner, 2016). We adopt this concept of novelty-induced plasticity modulation for our goal of local, rapid adaptation. We mimic a novelty-induced dopamine release by including a *dopaminergic neuron* at a given layer of a CFN (see Figure 2). We discuss how to identify novel information in an STDP-trained SNN, how the dopaminergic neuron is designed to fire under those conditions, and how the dopaminergic neuron modulates plasticity.

In STDP-trained SNNs, the weight vectors stabilize on the radial center of seen input clusters and are more likely to fire for inputs to which they are *angularly closer*–meaning inputs where the angle between the input vector and the weight vector are smaller for a given vector magnitude (see sections 2.3.4.1, 2.4.2.4). In other words, a sample from an unseen distribution will be less likely to induce firing than a sample from a learned distribution. Thus, when an input sample results in little-to-no firing activity at a given layer of neurons, we may assume that it contains information novel to that layer. (Data in Figure S3 in the Supplementary Material validate this assumption, showing that a dopaminergic neuron designed to fire under these conditions is indeed triggered more frequently whenever the system switches to an unseen class and otherwise sees a reduction in triggered dopaminergic activity as the new class is learned over time. See Supplementary Section S3 for more details).

We design the dopaminergic neuron with a resting potential higher than its firing potential, giving it a self-firing property. It is additionally suppressed via inhibitory connections from the other neurons in its layer so that it only spikes when they do not. This setup allows the dopaminergic neuron to fire only when novel information is detected.

**Figure 3:**
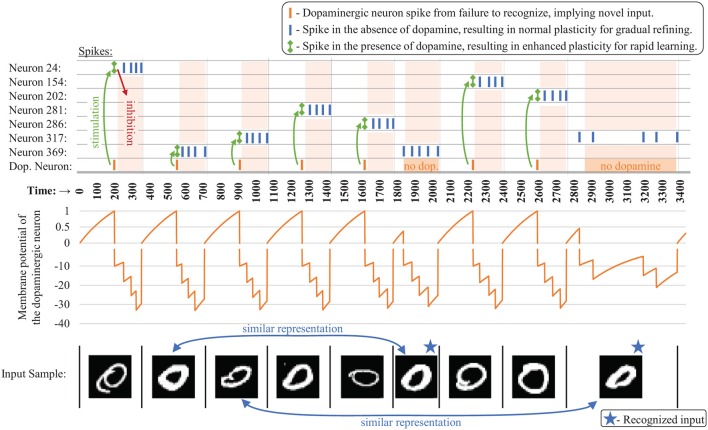
Example spiking activity **(top)** showing the interaction between the dopaminergic neuron and the other neurons during training for the first several samples **(bottom)** for a CFN of 400 neurons. Only neurons that fired during this small time interval are shown. The membrane potential of the dopaminergic neuron is shown **(middle)**, demonstrating its self-firing property unless inhibited, with a firing threshold at 1 (A break is shown in the graph at zero indicating a different scale for the positive and negative values).

When it fires, the dopaminergic neuron enhances plasticity by temporarily boosting the learning rate of the other neurons in its layer all the way to one while simultaneously stimulating firing in those other neurons via excitatory synaptic connections that we are calling *dopaminergic weights*. Because of the lateral inhibition discussed previously, once one of the stimulated neurons fires, it prevents or reduces the probability of the other neighboring neurons from firing. A neuron with a boosted learning rate then resets its learning rate the next time it fires or receives an inhibitory signal from a neighboring neuron, indicating that one of its neighbors has fired. Thus, while the dopamine signal is sent to many neurons, only the first neuron(s) to fire undergo the enhanced plasticity, creating heterogeneous plasticity and allowing the dopamine signal to perform an isolated targeting for local, rapid adaptation rather than global interference. Temporarily modulating the learning rate to the full value allows the first neuron that responds during a dopamine release to undergo a one-shot rapid learning of the current, novel sample and be “reassigned” without corruption from its old weight values. Then the learning rate is reset, allowing the representation to generalize with traditional, gradual weight changes. Figure 3 presents an example of the dopaminergic neuron in operation. The dopaminergic neuron fires for novel representations and does not fire if an input is similar to one already seen.

Due to the rapid learning that occurs in the presence of dopamine and the lack of traditional homeostatic threshold dynamics, we also modify the STDP learning rule for improved stability, discussed later in more detail in section 2.4.2.2.

#### 2.2.2. Targeted Stimulation for Controlled Forgetting via Trained Dopaminergic Weights

We have addressed how to make the forgetting process rapid and local in order to reduce interference between old and new information. However, we must also control the specific locality of the forgetting so as to maintain high accuracy for previous tasks. A uniform stimulation would cause the neuron that is angularly closest to the input to fire first and adapt to the novel information, independent of how useful that neuron is for previous tasks. When the network is refining representations that have already been seen, adjusting the closest weight vector is appropriate to promote generalization. However, when novel data in presented in high-dimensional space, the closest neuron is more likely to be one that has already learned a distribution from a previous class rather than an unused neuron that is completely uncorrelated. Thus the stimulation must be controlled to avoid overwriting the most essential information from previous tasks. We provide this control by heterogeneously stimulating the other neurons to fire during the release of dopamine via the excitatory dopaminergic weights. Training these weights allows specific neurons to be targeted to undergo forgetting and re-learning.

To minimize accuracy degradation caused by forgetting, we would ideally like to forget outlier or stale information rather than commonly-used or recent information that may be essential for returning to previous tasks, applying knowledge from old tasks to new tasks, or generalizing the rapidly learned novel information. As a proxy for this categorization, we target neurons with low overall firing frequency (outliers) or less recent firing activity (stale). Considering firing age over firing frequency is a tunable parameter that controls how much if any preference should be given to more recent tasks. For the experiments in this paper, we consider all tasks as equally important no matter how recently seen, so we target neurons with low firing frequency.

For these purposes, we enact a simple local learning rule: a dopaminergic weight depresses each time its post-synaptic neuron fires. This rule causes a dopaminergic weight to be smaller when the post-synaptic neuron it is targeting has a higher firing rate, and vice versa. To maintain positive values, the depressions are proportional to the current value, causing an exponential decay. Otherwise, the dopaminergic weights experience a gradual potentiation. Potentiation must occur to prevent the weights from tending toward zero with differences between weights too small to distinguish on implementations with finite precision. The rate of potentiation is irrelevant in our setup as long as it is the same for all dopaminergic weights in the layer, maintaining their relative values, because the dopaminergic neuron continues to send the dopaminergic signal until one of the other neurons in the layer fires. For the experiments in this work, we effect this potentiation by L2-normalizing the fan-out vector of dopaminergic weights after a depression.

### 2.3. Models

In this subsection, we describe the input, synapse, and neuron models and associated probability distributions that are useful in selecting the appropriate hyperparameters for unsupervised lifelong learning.

#### 2.3.1. Input Encoding

Input samples are encoded as Poisson spike trains, following the mathematical model of a Poisson point process (PPP), where the spike rate λ_*i*_ of an input neuron is proportional to the pixel intensity of input *i*. Thus, the number of spikes in a given time window follows the distribution of a Poisson random variable with an expectation proportional to the input value. For perception tasks on static images, there is no temporal information in a single sample, and thus rate encoding is one of the most common encoding methods for SNN image perception implementations as it maintains statistical independence between individual input spikes, which is useful for the computationally less expensive one-sided STDP curve, discussed later in section 2.4.2^1^.

Each spike is modeled as a time-shifted delta function. The precise time of the *kth* most recent spike from input *i* is represented as *t*_*ik*_. Being a PPP, the timing between two sequential spikes on a given input channel are drawn from an exponential random distribution, also with rate λ_*i*_. The time passed since the *kth* most recent spike from *i* at time *t* is represented as *t*_|*ik*|_ = *t* − *t*_*ik*_ and follows the distribution of a gamma random variable *T*_|*ik*|_ ~ *gamma*(α = *k*; β = λ_*i*_). The vector of all input rates for each dimension of the given sample is represented as $\vec{\lambda }$.

#### 2.3.2. Synapse Model

We model the synaptic connections between neurons as a multiplicative weight which is applied to the delta spike from its pre-synaptic neuron and then added to the membrane potential of its post-synaptic neuron, creating a exponential kernel response^2^. We represent the weight of the synapse connecting input *i* to neuron *j* as *w*_*ij*_ and the vector of all inputs to neuron *j* as ${\vec{w}}_{j}$.

#### 2.3.3. Spiking Neuron Model

We use the common Leaky-Integrate-and-Fire (LIF) neuron model, in which a neuron's membrane potential *v*_*mem*_ undergoes a continuous decay according to the differential equation in (1), where τ_*mem*_ is the membrane decay constant and *v*_*rest*_ is the resting potential. The membrane potential is also potentiated or depressed by incoming excitatory or inhibitory signals, respectively. If the membrane potential reaches or surpasses the neuron's firing threshold *v*_*th*_ then the neuron fires, producing an output spike and resetting its potential to *v*_*reset*_. Without loss of generality, we set *v*_*rest*_ to zero as a reference voltage. For model and evaluation simplicity, we also set *v*_*reset*_ to zero and have no refractory periods.

(1) $$\begin{array}{r} {\overset{∙}{v}}_{mem}=\frac{-\left(v_{mem}-v_{rest}\right)}{\tau_{mem}} \end{array}$$

#### 2.3.4. Membrane Potential Distribution

To estimate the relative firing distributions of competing LIF neurons, it is useful to understand the distribution of their membrane potentials. Assuming a firing event has yet to occur, the effect of a Poisson spike train on a neuron's membrane potential with exponential leakage may be viewed as a shot-noise process (Hohn and Burkitt, 2001). A Poisson spike train from input *i* is the summation of many spikes represented as delta functions:

(2) $$\begin{array}{r} N_{i}=\underset{k}{\sum }\delta_{T_{\left|ik\right|}} \end{array}$$

This stochastic process produces the following pre-firing membrane potential induced on neuron *j* by the spike train from input *i*:

(3) $$\begin{array}{r} V_{ij}\left(t\right)=\int f_{ij}\;\left(t\right)\;N\left(dt\right)=\underset{k}{\sum }f_{ij}\;\left(t-T_{k}\right), \end{array}$$

where $f_{ij}\left(t\right)=w_{ij}e^{-t/\tau_{mem}}$. The Laplace transform of this shot-noise process is:

(4) $$\begin{array}{r} L\left(\theta \right)=E\left[e^{-\theta V_{ij}\left(t\right)}\right]=e^{g\left(\theta \right)} \end{array}$$

where $g\left(\theta \right)=\lambda_{i}\int_{0}^{t}\left(e^{-\theta f_{ij}\left(v\right)}-1\right)dv$.

##### 2.3.4.1. Mean pre-firing membrane potential

The 1^*st*^ moment, which is the mean pre-firing potential caused by input channel *i*, is given by:

(5) $$\begin{array}{l} E[V_{ij}(t)]=-{\left[\frac{dℒ(\theta )}{d\theta }\right]}_{\theta =0}=-{\left[\frac{de^{g(\theta )}}{d\theta }\right]}_{\theta =0}\\ \;=-{\left[e^{g(\theta )}\right]}_{\theta =0}{\left[\frac{dg(\theta )}{d\theta }\right]}_{\theta =0}\\ \;=-\lambda_{i}[\int_{0}^{t}(-f_{ij}(v)e^{-\theta f_{ij}(v)})dv{]}_{\theta =0}\\ \;=\lambda_{i}\int_{0}^{t}f_{ij}(v)dv=\lambda_{i}w_{ij}\tau_{mem}(1-e^{-t/\tau_{mem}}) \end{array}$$

For all inputs, represented as the rate vector $\vec{\lambda }$, the mean combined pre-firing potential of neuron *j* is:

(6) $$\begin{array}{l} E\left[V_{j}\left(t\right)\right]=\tau_{mem}\underset{i}{\sum }\lambda_{i}w_{ij}\left(1-e^{-t/\tau_{mem}}\right)\\ \;=\tau_{mem}\left(\vec{w_{j}}•\vec{\lambda }\right)\left(1-e^{-t/\tau_{mem}}\right) \end{array}$$

In steady-state this converges to: $\tau_{mem}\left(\vec{w_{j}}•\vec{\lambda }\right)$, which is important for discussions later in sections 2.4.2, 2.4.3.1.

##### 2.3.4.2. Variance of pre-firing membrane potential

Continuing to the second moment, we can calculate the variance of the pre-firing membrane potential that is induced on neuron *j* by incoming spikes received from input *i*:

(7) $$\begin{array}{l} Var(V_{ij}(t))=E[V_{ij}{\left(t\right)}^{2}]-E{\left[V_{ij}\left(t\right)\right]}^{2}\\ \;={\left[\frac{d^{2}ℒ(\theta )}{d\theta^{2}}\right]}_{\theta =0}-E{\left[V_{ij}\left(t\right)\right]}^{2}\\ \;={\left[e^{g(\theta )}(g^{\prime }{\left(\theta \right)}^{2}+g^{\prime \prime }(\theta ))\right]}_{\theta =0}-E{\left[V_{ij}\left(t\right)\right]}^{2}\\ \;=E{\left[V_{ij}\left(t\right)\right]}^{2}+\lambda_{i}\int_{0}^{t}f_{ij}{\left(v\right)}^{2}dv-E{\left[V_{ij}\left(t\right)\right]}^{2}{}^{2}\\ \;=\frac{1}{2}\lambda_{i}\tau_{mem}w_{ij}^{2}(1-e^{-2t\tau_{mem}}) \end{array}$$

The combined variance of the potential induced by all inputs is:

(8) $$\begin{array}{l} Var\left(V_{j}\left(t\right)\right)=\frac{1}{2}\tau_{mem}\underset{i}{\sum }\lambda_{i}w_{ij}^{2}\left(1-e^{-2t\tau_{mem}}\right)\\ \;=\frac{1}{2}\tau_{mem}\left(\vec{\lambda }•{\vec{w_{j}}}^{\circ 2}\right)\left(1-e^{-2t\tau_{mem}}\right) \end{array}$$

where ${\vec{w_{j}}}^{\circ 2}$ represents the Hadamard square of the weight vector. This equation is important for discussions later in section 2.4.2.2.

### 2.4. Experimental Methodology

To evaluate the effectiveness of our proposed lifelong learning approach, we simulated CFNs on the MNIST dataset (Lecun et al., 1998) on network sizes of 400, 900, 1,600, 2,500, 3,600, 4,900, and 6,400 excitatory neurons, each for five different seeds. We compare the CFNs that have dopaminergic neurons to the same setups without dopaminergic neurons, both with and without homeostasis from adaptive thresholding. This section details the experimental methodology of the simulations for the CFNs and the comparison networks.

#### 2.4.1. Simulation Setup

Each network was evaluated on the MNIST dataset for two different scenarios: (1) *interleaved* classes where classes are distributed uniform randomly, providing the network with samples from each class throughout the entire training process; and (2) *disjoint* classes where all samples from one digit are presented before moving to the next digit and never returning to previous digits after changing classes. The first scenario is meant to represent traditional offline training in which all training data is already available. The second scenario is meant to test lifelong learning by representing a changing environment with samples presented in the worst-case possible ordering–entirely sequential. In both scenarios, class labels are not provided during training. This means that the network receives no external indication of when a task/digit change occurs in the disjoint scenario.

Other than sequentializing the MNIST dataset in the disjoint scenario, our training and testing procedure follows closely with that of Diehl and Cook (2015), who demonstrated competitive unsupervised STDP training on MNIST in the traditional interleaved scenario.

##### 2.4.1.1. Training process

In both training scenarios, samples are presented one-by-one to the network. For the current sample, input neurons fire at the sample rate until the system registers at least five output spikes, as followed by Diehl and Cook, which is generally enough to confidently identify the input in view of the stochasticity in the SNN.

In contrast to Diehl and Cook, if a given sample does not produce enough output spikes we do not continue to increase the input firing rate during training on the CFNs, since the dopaminergic neuron takes care of stimulating neurons in the absence of a good match. After five output spikes are registered, all membrane potentials are reset to avoid one sample interfering with the next, and then the next sample is presented. Details of the STDP learning rule implementation are provided in section 2.4.2.

##### 2.4.1.2. Testing process

In the disjoint scenario, we measure effective lifelong learning over time by evaluating each network after each task change to determine its current accuracy for all classes seen up to that point. Networks in the interleaved scenario are only evaluated at the end of the training process. During evaluation we pause learning and freeze network parameters to prevent samples from older classes or samples from the testing set from affecting the network.

As training is performed entirely without supervision and without knowledge of a task change, the final network outputs must be assigned class labels for evaluation. While the network is frozen, label assignment is done by inference on the training set, followed by evaluation on the testing set. The MNIST dataset is already highly clustered in its input space, and therefore a supervised linear classifier is already capable of competitive accuracy. Because of this, no final linear readout classification layer is added to avoid the label assignment process acting as a traditional supervised linear classifier. Instead, following the unsupervised evaluation method of Diehl and Cook, each trained neuron is *directly* assigned a class label and no linear combination of these neuron outputs is performed. Rather, the class decision is winner-take-all, choosing the class of the neuron that spiked the most for that sample. As a control, we also perform this same label assignment and evaluation process on networks with randomized weights, also averaged over five seeds, to compare with the accuracy achievable solely by this label assignment process.

With frozen parameters, dopaminergic adaptation does not occur during label assignment and testing set evaluation. Instead, for inference a poorly-recognized input is assigned the class of the closest trained neuron by continuing to increase input firing rates until a sufficient response is recorded as is done in the other networks.

##### 2.4.1.3. Event-driving computation

Using exponential kernels, we treat spikes as inducing instantaneous voltage potentiations in the respective post-synaptic neuron membranes with exponential decay. As such, neurons only fire upon receiving an incoming spike and will not fire between incoming spikes, with the exception of the dopaminergic neurons which are handled separately. This allows us to emulate the networks using purely event-driven computation rather than breaking time into discrete time steps and updating neurons states at each time step. Because we encoded input spike trains as Poisson point processes, the time between spikes is an exponential random variable with λ_*i*_ = *input*_*i*_. Therefore, rather than incrementing time in fixed intervals, we calculate the time until the next input spike arrival and decay all the traces and membrane potentials according to that time interval before processing that input spike.

The dopaminergic neurons are an exception, as they fire in the *absence* of input spikes. Therefore, before processing an input spike, we first check to see if the dopaminergic neuron would have fired earlier, in which case, it is processed at its respective time interval first.

#### 2.4.2. STDP Learning

STDP's Hebbian learning rule involves potentiation or depression of a synaptic weight based on the timing of firing events. This section details how it is implemented and modified for these experiments.

##### 2.4.2.1. One-sided STDP

As the input information in our system is encoded only in the spike rate, we can employ the computationally less-expensive one-sided version of STDP, evaluated at the post-synaptic firing event:

(9) $$\begin{array}{r} \Delta w=\alpha \left(pre-offset\right) \end{array}$$

where α is the learning rate, *pre* is a trace of pre-synaptic firing events, and *offset* is the value to which the pre-synaptic traces are compared, determining potentiation or depression.

The *pre* trace follows a similar distribution as the membrane potential (see section 2.3.4), only with a different time constant and without being weighted by the synapse, and so its expected value is also proportional to the input spike rate (e.g., *E*[*pre*_*i*_] = λ_*i*_τ_*pre*_). Correlated potentiations in the direction of $p\vec{r}e$ therefore provide Hebbian learning by angularly migrating $\vec{w}$ toward the angle of the input vector $\vec{\lambda }$. Anti-Hebbian depression reduces weights from uncorrelated inputs and is provided by subtracting the *offset* term for one-sided STPD rather than performing additional weight processing at pre-synaptic firing events.

##### 2.4.2.2. Stabilizing STDP

**Figure 4:**
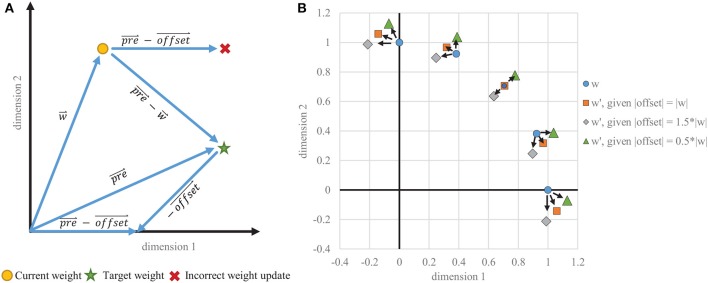
Instability of one-sided STDP. **(A)** Example vectors showing how a static offset does not result in a correct weight change. The goal is to migrate $\vec{w}$ toward the $p\vec{r}e$ trace, which is proportional to the input $\vec{\lambda }$. The $of\vec{f}set$ vector that is subtracted from $p\vec{r}e$ must be dynamically tied in each dimension to $\vec{w}$, rather than being the same in every dimension. **(B)** Weight change results for various starting positions where the target vector is equal to the current weight vector, which would ideally result in no weight change. With a static offset in each dimension, even scaled to the appropriate magnitude, the weight vectors do not stabilize on the target and instead migrate toward the axes, creating binarized weights when capped at zero.

Typically, *offset* is a constant value identical across all dimensions and can be thought of as a scaled ones vector, applying uniform anti-Hebbian depression. Such uniform depression does not, however, create a weight change in exactly the direction desired (see Figure 4) and causes instability in the STDP learning rule. This instability is usually controlled by adaptive thresholding and weight capping via exponential weight-dependence.

However, our CFNs with rapid one-shot dopaminergic learning of novel inputs cannot use such gradual approaches to stabilize. We provide the required stability to this STDP learning rule by correcting the direction of the weight change. Rather than a constant offset, we dynamically tie *offset* to the current weight value, which is an adaptation based on Oja's rule (Oja, 1982). To place *pre* and the weight on the same scale, we scale the pre-synaptic trace by the inverse of its decay rate τ_*pre*_, changing (9) to:

(10) $$\begin{array}{r} \Delta w=\alpha (\frac{pre}{\tau_{pre}}-w) \end{array}$$

This corrected weight change allows our CFNs to rapidly and accurately capture information from novel inputs during dopaminergic learning and otherwise gradually stabilize on the center of the cluster of input samples for which it has fired. Additionally, the stochasticity of the presynaptic trace can allow the values of some dimensions to significantly overshoot or undershoot their mean. Because of the rapid learning in the presence of dopamine, we capped each individual weight between 0 and 0.2 before normalization to prevent the outliers from distorting the normalization.

##### 2.4.2.3. Modulating STDP

Dopaminergic modulation of plasticity is implemented by dynamically changing the learning rate α. During normal operation, α is set to 0.01 for gradual generalizing refinement of the synaptic weights. When the dopaminergic neuron fires, α is temporarily set to one for the reasons discussed in section 2.2.1.

##### 2.4.2.4. Normalization

The MNIST dataset is a magnitude insensitive dataset, meaning that increasing or decreasing the intensity of a sample does not alter its class and that angular distance is more important than Euclidean distance. As given in (6), the mean pre-firing potential of a spiking neuron is proportional to the L2-norm of its weight vector and also to the L2-norm of the input rate vector. Although a larger mean pre-firing potential does not always correspond to a larger firing rate due to differing variances caused by the Hadamard square of the weight vector as shown in (8), the correlation between *E*[*V*] and the firing rate sufficiently holds for datasets like MNIST with inputs of large enough dimensions and fairly comparable input sparsity between samples.

As such, for a given input and assuming equal weight vector magnitudes, the neuron that is angularly closest to the input will be more likely to fire, allowing for unsupervised Hebbian learning by training neurons on correlated inputs. Therefore, we L2-normalize each neuron's weight vector, and for the same reason the input rate vectors are also L2-normalized. Weight normalization has recently been shown to occur in biology (El-Boustani et al., 2018) and may still be considered a localized function, as the processing can occur at the post-synaptic neuron to which all the weights in a given weight vector are directly connected.

#### 2.4.3. Timing and Time Constants

As our evaluations and simulations are purely event-driven, the concept of discrete computational time steps is not applicable. Timing parameters are thus purely relative. Therefore, without loss of generality, the L2 normalized input rate vectors were defined as having an L2 rate magnitude of one spike per time unit, and all other timing values are relative to that. This subsection discusses the timing values used in the simulations.

##### 2.4.3.1. Membrane decay time constant

According to Equation (6), the expected value of the membrane potential saturates in time according to (1−*e*^−*t*/τ^). A smaller τ results in a faster convergence to the steady state, or, equivalently, fewer input spikes to converge. E.g., in five time constants, the expected potential reaches over 99% of is steady-state value. However, using (8), the steady state standard deviation of the potential in proportion to the mean decreases as the decay rate increases:

(11) $$\begin{array}{r} \frac{\sqrt{Var\left(V\right)}}{E\left[V\right]}\propto \frac{1}{\sqrt{\tau }} \end{array}$$

Thus, a larger membrane decay constant is better for proper discrimination between two differing inputs, but increases the number of computations. For the L2-normalized MNIST dataset with 784 input dimensions, the angular distances between samples of differing classes are close enough to require at least 10 to 15 normalized time units for τ_*mem*_ in order to successfully establish a firing threshold that can discriminate between classes, and so τ_*mem*_ was set to 15 time units.

##### 2.4.3.2. Time to recognize

A τ_*mem*_ of 15 still produces enough variance according to (8) that two to three time constants (between 30 and 45 time units) is on average sufficient time for the potential to rise above its steady-state mean. As mentioned earlier, we identify successful recognition of an input sample after registering five output spikes. Therefore, a total of 150–225 time units was generally sufficient to produce five sequential firing events in a reference vector neuron with a center close to the input.

In our simulations, we found little accuracy change by adjusting this hyperparameter within this range as long as the threshold voltage was appropriately tuned, so we fixed the time to recognize at 200 normalized time units for each simulation. We tuned the dopaminergic neuron to fire after those 200 time units unless it has been otherwise inhibited as discussed in section 2.2. Specifically, with *v*_*reset*_ set to zero as a reference voltage, the dopaminergic neuron's firing threshold *v*_*th*_ was set to one with a resting voltage set higher at two, causing the membrane potential to rise until it fires. Setting its rising time constant to $\frac{200}{ln\left(2/1\right)}$ then meets this objective. Figure 3 shows the membrane potential of the dopaminergic neuron during simulation for the first several samples as an example of its operation over time.

We also set τ_*pre*_ to the same timing value of 200 time units to capture as much of the input train as possible because of the rapid one-shot dopaminergic learning of novel samples.

#### 2.4.4. Determining *v*_*th*_ Without Adaptive Thresholding

As discussed in section 2.1.2, adaptive thresholding for homeostasis can interfere with lifelong learning on changing input distributions by temporally and spatially distributing the firing activity. Long-term adaptive thresholding may still be used with controlled forgetting if properly tuned, but our proposed method of enhanced plasticity and stimulated firing of infrequently-firing neurons is itself a form of deliberate, controlled homeostasis. Therefore, for a more accurate evaluation of the CFNs, we do not have the CFNs employ any adaptive thresholding–having static thresholds instead. With normalized weight vectors and input vectors, the larger the ratio *v*_*th*_:*E*[*V*(*t*)] the closer the input rate vector must be angularly to the weight vector to produce a given firing probability. Determining the proper *v*_*th*_ without dynamic adaptation, therefore, depends on the tightness of the clustering in the dataset. With this context, we included *v*_*th*_ in our hyper-parameter search, discussed next.

#### 2.4.5. Hyper-Parameter Sweep

SNNs are known to be highly sensitive to hyper-parameters, especially during unsupervised learning without error signals to provide dynamic corrections. We perform a small search in the hyper-parameter space, adjusting *v*_*th*_ and the number of training epochs. Results from this search are shown in Table 1, with hyperparameters resulting in the best accuracy highlighted for each size. Good machine learning practice requires that we choose the system parameters based only on the training set, so only training set accuracy results are shown here. Testing accuracy results are discussed later in the section 3. A similar hyper-parameter sweep was performed for the non-dopaminergic SNNs that also do not have homeostatic adaptive thresholding, as well as for the SNNs with randomized weight vectors.

**Table 1:** Training accuracy results of hyper-parameter sweep for each network size across both *v*_*th*_ and number of training epochs per task.

		**# of training epochs per task**			
**Neurons**	**v_*th*_**	**1 (%)**	**5 (%)**	**10 (%)**	**20 (%)**
400	13.50	87.63	83.82	79.23	74.15
	13.75	87.63	84.07	78.34	75.18
	14.00	86.64	82.49	77.11	75.20
	14.25	85.50	83.59	75.75	75.06
900	13.50	89.78	91.07	89.36	85.31
	13.75	89.11	90.91	89.81	86.24
	14.00	87.83	91.47	89.71	84.75
	14.25	86.82	91.46	89.94	84.14
1,600	13.50	91.54	92.42	92.21	91.35
	13.75	91.24	92.87	92.48	91.40
	14.00	90.08	93.34	92.20	91.30
	14.25	88.48	93.06	92.85	91.74
2,500	13.50	93.15	93.62	93.46	93.13
	13.75	92.82	93.65	94.06	93.20
	14.00	91.80	93.37	94.28	93.53
	14.25	90.06	93.18	94.04	93.49
3,600	13.50	93.88	94.09	94.04	93.80
	13.75	93.90	94.12	94.48	94.52
	14.00	93.31	94.02	94.53	94.52
	14.25	92.33	93.27	94.40	94.27
4,900	13.50	94.51	94.91	94.67	94.77
	13.75	95.00	94.92	94.82	95.09
	14.00	94.61	94.85	94.97	95.21
	14.25	93.59	93.82	94.54	95.29
6,400	13.50	95.39	95.25	95.28	95.25
	13.75	95.42	95.55	95.39	95.68
	14.00	95.33	95.59	95.42	95.79
	14.25	94.79	94.99	95.21	95.88

*Highlighted cells are best configuration for each size*.

##### 2.4.5.1. Neuron firing thresholds, *v*_*th*_

Based on the discussion above, *v*_*th*_ should be close to but slightly less than τ_*mem*_ in voltage units, which is set to 15 time units. For MNIST, we initially found that if *v*_*th*_ is much less than 13.5, a neuron may too likely fire for samples from other classes, while if *v*_*th*_ is much higher than 14.25, a neuron may not fire for very close samples, even different stochastic instances of the same sample. We therefore tested each setup with four different threshold values in this range: 13.5, 13.75, 14.0, and 14.25. Smaller networks require each individual neuron to capture a larger subset of input samples, generally requiring slightly lower thresholds than those in larger networks.

##### 2.4.5.2. Number of training epochs

Larger networks can capture representations that are less common but still useful. As such, for larger networks more epochs within a class are required before proceeding to subsequent tasks in order to refine the less common representations. For smaller networks, on the other hand, more epochs may reinforce less useful outliers, making it more difficult to make room for subsequent tasks.

#### 2.4.6. Comparison of *E*[*V*(*t*)] at *v*_*th*_ With K-Means Clustering Angular Error

**Figure 5:**
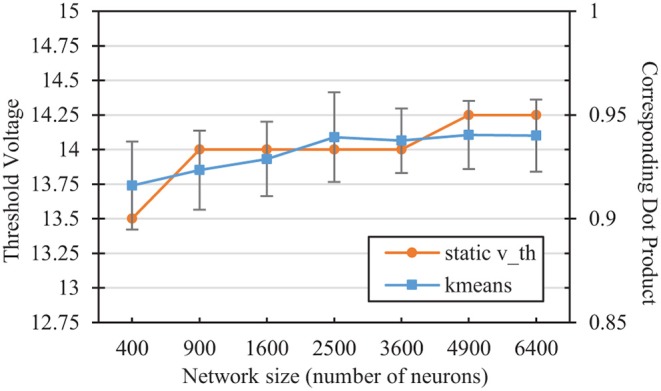
Comparison of the static *v*_*th*_ selected in the hyperparameter sweep with the corresponding dot product of the nearest training error in a kmeans network of the same size. The kmeans error bars represent two standard deviations over 100 trials each.

We can compare the *v*_*th*_ values selected in the hyper-parameter search with the mean angular distance to a neuron's weight vector that would on average result in a membrane potential equal to that threshold. Performing a simple k-means clustering on the L2-normalized MNIST dataset yields information on the relative desired scope of each reference vector, depending on the number of reference vector neurons. Figure 5 shows the dot product associated with the angular distance of the closest training sample/reference vector pair from differing classes for each network size after k-means clustering. The figure also shows the average membrane potential of a spiking neuron corresponding to these angles. For SNNs, neurons that are able to fire for samples that are further away than these angles are thus more likely to fire for samples of the wrong class. As the number of reference vector neurons increases, the portion of the input space per neuron decreases, improving accuracy by allowing each individual neuron to be more restrictive in its angular scope, which is relatively similar to those associated with the *v*_*th*_ values selected in the hyper-parameter sweep.

## 3. Results

In this section, we present the results of simulating the CFNs and the non-dopamine comparison networks for the various sizes in both the interleaved classes scenario and the fully disjoint classes scenario. We present both the combined accuracy and the per digit accuracy, with final results and (in the disjoint scenario) results throughout the attempted lifelong learning process.

### 3.1. Combined, Across-Task Accuracy Results

**Figure 6:**
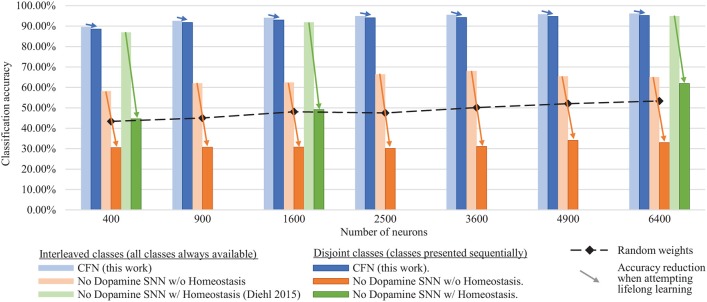
Final classification accuracy at various sizes of the CFNs compared to SNNs without dopamine. Accuracy is shown for both the interleaved class scenario and the disjoint class scenario, showing the resulting accuracy reduction by sequentializing the classes. CFNs show average over five seeds.

Figure 6 shows the final combined, across-task classification accuracy of the CFNs and comparison networks for both the interleaved scenario and the disjoint scenario for all network sizes. The comparison with Diehl and Cook (2015) is provided for the network sizes for which results were published (400, 1,600, and 6,400). In the fully disjoint scenario, the 6,400 CFN achieves on average 95.24% classification accuracy across all digits, compared to 32.97% for a non-dopamine SNN without homeostasis, 61.95% accuracy for a non-dopamine SNN with homeostasis, and 53.30% accuracy for an SNN with random weights (Per-neuron activity statistics are available in the Supplementary Material).

**Figure 7:**
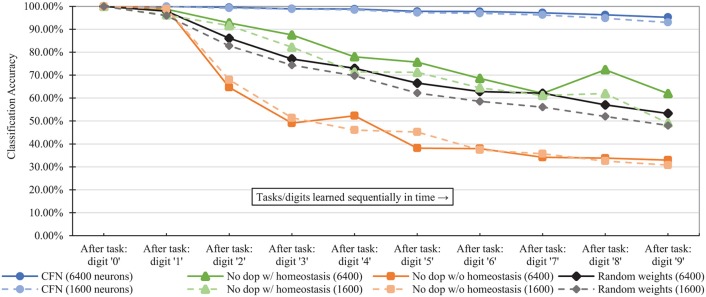
Classification accuracy over time at each stage of the learning process (i.e., after each new task/digit) in the disjoint scenario, comparing the proposed CFNs to SNNs without dopamine and to the randomized weight control. Accuracy is for all previous tasks, up to and including the current task. CFN results are averaged over five seeds.

Figure 7 shows the combined, across-task accuracy over time for the CFNs and comparison networks for network sizes of 1,600 and 6,400 neurons. (CFN results for the other sizes are available in the Supplementary Material). The combined, across-task accuracy over time is defined as classification accuracy on the portion of the testing set consisting of all previously-seen classes, up to and including the current task. For the 6,400 size, the CFN incurs its largest accuracy drop at the last stage, adding digit ‘9,' dropping 1.06 percentage points. In comparison, at that size the non-dopamine SNN without homeostasis incurs a 34.41 percentage point drop when adding digit ‘2,' the non-dopamine SNN with homeostasis incurs a 10.41 percentage point drop adding digit ‘9,' and the SNN with random weights incurs an 11.82 percentage point drop adding digit ‘2.'

### 3.2. Per-Digit Accuracy Results

**Figure 8:**
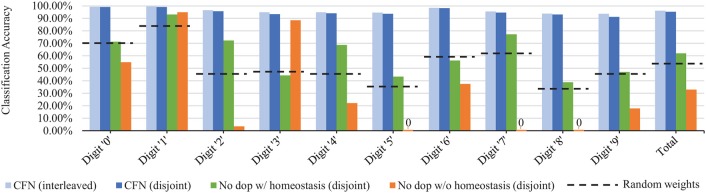
Final per-digit accuracy (size 6,400), comparing interleaved CFN accuracy to the disjoint CFN accuracy. Also showing failure for individual digits in the disjoint scenario for SNNs without dopamine.

Figure 8 shows the final accuracy of each individual task/digit by the end of the training process for 6400 neurons, comparing the distribution of accuracy across tasks for the CFNs in both the interleaved and disjoint scenarios, as well as with both the non-dopamine SNNs in the disjoint scenario and the randomized weights. In the disjoint scenario, the CFN's final worst performing class is digit ‘9' at 91.18% accuracy, which is also the worst performing class in the interleaved scenario at 93.60% accuracy. In comparison, for the other networks in the disjoint scenario, the final worst performing class is digit ‘8' at 38.81% accuracy for the non-dopamine SNN with homeostasis; digits ‘5,' ‘7,' and ‘8' tied at 0.00% accuracy for the non-dopamine SNN without homeostasis; and digit ‘8' at 33.37% accuracy for the SNN with random weights.

**Figure 9:**
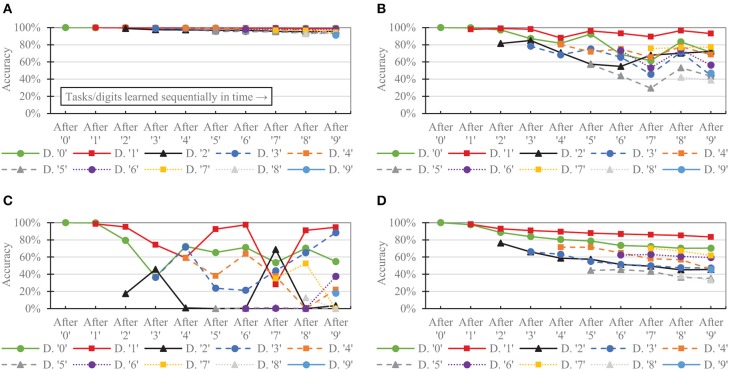
Per-task/digit classification accuracy as new tasks/digits are added over time for the following networks, all of size 6,400: **(A)** the proposed CFN **(B)** no dopamine SNN with homeostasis **(C)** no dopamine SNN without homeostasis, **(D)** SNN with random weights.

Figure 9 shows the per-digit accuracy over time for each network of 6,400 neurons. (Per-digit false positives over time are provided in the Supplementary Material). The CFN incurred its largest per-digit accuracy drop for digit ‘4' after adding digit ‘9,' decreasing 3.89 percentage points for digit ‘4' during that task change. In comparison, the non-dopamine SNN with homeostasis incurred a 23.07 percentage point drop for digit ‘4' at that same transition; the non-dopamine SNN without homeostasis incurred a 69.52 percentage point drop for digit ‘1' after adding digit ‘7,' and the SNN with random weights incurred a 10.98 percentage point drop in accuracy for digit ‘4' when adding digit ‘9.'

## 4. Discussion

In this section, using a qualitative analysis we discuss reasons why the non-dopamine SNNs failed at lifelong learning in the disjoint scenario and how the CFNs avoided those failures. We also discuss the expected sequential penalty and graceful degradation of accuracy.

### 4.1. A Qualitative Analysis

**Figure 10:**
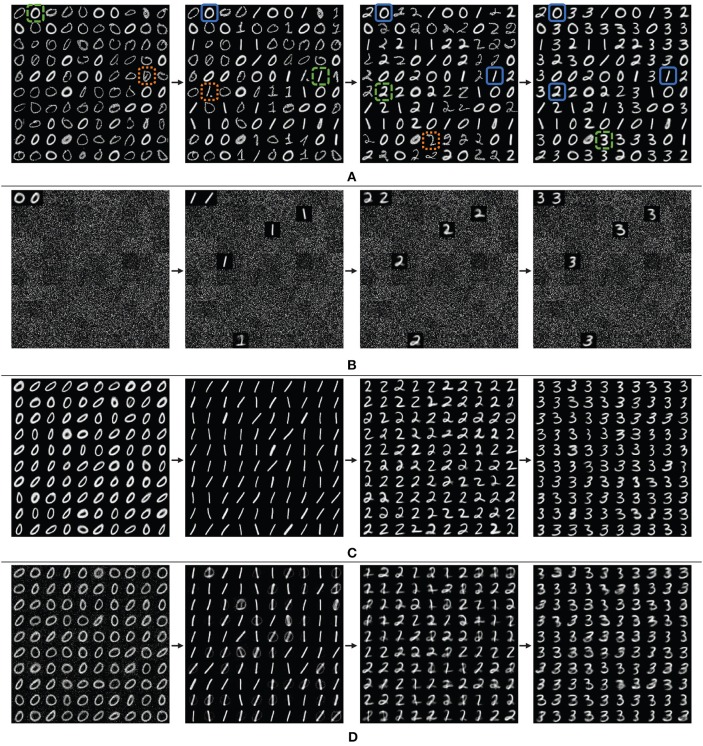
Grid view of the weight vectors of reference neurons over time, showing the first four digits, learning ‘0' through ‘3' for **(A)** the proposed CFN, **(B)** a non-dopamine SNN without homeostasis, and **(C)** a non-dopamine SNN with homeostasis, each with 400 neurons, although only the 100 top-firing neurons are shown for space. For the CFN, digits highlighted in dashed green are examples of successfully learned generalized representations. Digits highlighted in dotted orange are examples of outlier representations. Digits highlighted in solid blue are examples of representations preserved from previous tasks. Also shown is **(D)** another non-dopamine SNN with homeostasis, but with reduced learning on each digit, showing catastrophic interference between classes causing corruption.

In these fully-connected one-layer SNNs, each neurons weight vector can be viewed as a reference vector that captures a specific input representation, ideally successfully generalized. As such, we may qualitatively observe the success of dopaminergic learning over time by viewing these representations. For a better visual demonstration of the disjoint scenario, we show the weights of the networks for the first four digits ‘0' through ‘3' in Figure 10, with 100 neurons arranged in a 10x10 grid.

Note that in the CFN case (Figure 10A) there are two very distinct categories of representations. The digit representations that appear to have a more consistent pixel intensity and a more consistent line width and curvature are generalized representations refined by many similar samples in a cluster. On the other hand, the digit representations that appear less defined and with more irregularity in pixel intensity are outlier representations from only one or a few samples. Notice that the digit representations that are preserved from one task to another are the useful generalizations rather than the outliers, which on the other hand are the first to be overwritten when space for a new task is required. In addition, the representations that are preserved from previous tasks experience very little and infrequent corruption during later learning stages. The dopamine signals are able to successfully replace old information with new information without interference and while maintaining accuracy because of the targeted localization.

In contrast, we can visually see the failure of the non-dopamine SNNs in the disjoint scenario. In the network without homeostasis (Figure 10B) we see that only a few neurons experienced any learning. Without homeostasis the neurons that fired first migrated closer to the input distributions and dominated the firing activity. Even when the input distribution changed between tasks, the already used neurons were closer to the new distributions than the unused neurons with random weight vectors. Continuing the reuse the same neurons caused the SNN to overwrite and forget previous tasks.

Next, in the network with homeostatic adaptive thresholding (Figure 10C), we see a better use of network resources from the distributed firing activity. But without targeted dopaminergic modulation homeostasis distributes the learning for a new task over all the neurons previously used in earlier tasks. Even when the learning per-digit is reduced (Figure 10D), the activity for the new tasks are still globally distributed by the adaptive thresholding, causing corruption between tasks.

The CFNs with dopaminergic learning avoid globally distributing firing activity during a single task by not having traditional homeostatic adaptive thresholding. In addition, the CFNs avoid continuing to reuse the same neurons by proactively identifying novel data and targeting specific neurons to learn the novel data, preserving essential information from previous tasks.

We note that for the failed networks where older classes are entirely overwritten by new classes, the networks still report some, albeit poor, accuracy for the forgotten tasks. This is because the varied intra-class distributions can still be somewhat useful at differentiating inter-class distributions. For this purpose, the accuracy comparisons to the SNNs with random weights are essential at identifying catastrophic forgetting, indicating that around 40–50% is a failure baseline for unsupervised learning using SNNs of these sizes on the MNIST dataset.

### 4.2. The Expected “Sequential Penalty”

We see that the CFNs in the disjoint scenario perform on par with the interleaved scenario, averaging only a 1.04% accuracy reduction across all sizes. This penalty is expected due to sequentializing the tasks. In fact, such a penalty may be impossible to completely avoid, as the interleaved scenario provides more information to the network throughout training by providing all distributions up front, whereas the disjoint scenario never provides an opportunity to temporally overlap learning of different distributions. Even so, the sequential penalty for the CFNs is minimal, and may be acceptable given the systems avoidance of catastrophic failure in the disjoint scenario. In fact, even with this penalty, the 6400 neuron CFN achieves a respectable 95.24% test accuracy after lifelong learning, which we believe is the best unsupervised accuracy ever achieved by a fixed-size, single-layer SNN on a completely disjoint MNIST dataset. The CFNs in the disjoint scenario even outperform (Diehl and Cook, 2015) in all cases for which they provide results, even though that work is in the interleaved scenario.

### 4.3. Graceful Degradation Instead of Catastrophic Forgetting

Controlled forgetting allows the network to gracefully degrade its accuracy in exchange for the ability to learn new tasks with limited resources, rather than failing. The true success of a lifelong learning system is shown not just by the final accuracy, but also by its performance throughout the training process and across training tasks. Notice how in Figure 8 while the system expectedly performs better for some tasks rather than others, there is no single task for which the system fails; i.e., the sequential penalty is spread between tasks. In fact, the lifelong system performs best at the same tasks (digits ‘0,’ ‘1,’ and ‘6’) and worst at the same tasks (digits ‘8’ and ‘9’) that the offline/non-lifelong system does.

We believe that this type of approach with modulated plasticity and targeted stimulation can be useful for allowing deployed systems to gracefully adapt to changing environments rather than failing to adapt or requiring frequent offline retraining.

### 4.4. Future Work

We expect that a deeper network will improve accuracy beyond that of these results and allow for learning of more complicated datasets. As mentioned earlier, in a deeper network, it may be that only the last few layers would require lifelong learning, performing a readout from a liquid state machine or a fixed feed forward network sufficiently pre-trained on low-level representations. We also plan to evaluate this method on time-encoded signals to improve sparsity and energy efficiency. Further, we hope to explore other dopaminergic weight adjustment policies that have a higher time-dependence or weight policies with habituation, such as in Panda et al. (2018), in order to allow for operation in an environment of changing priorities, and not just temporally separated tasks.

### 4.5. Conclusion

We presented a biologically-inspired dopaminergic modulation of synaptic plasticity to exploit STDP locality. Trained stimulation during the presentation of novel inputs allows the system to quickly perform isolated adaptation to new information while preserving useful information from previous tasks. This method of controlled forgetting successfully achieves lifelong learning. Our Controlled Forgetting Networks show only a slight reduction in accuracy when given the worst possible class ordering, i.e., completely sequential without revisiting previous classes, while successfully avoiding catastrophic forgetting.

## Data Availability Statement

The MNIST dataset used in this study can be found at http://yann.lecun.com/exdb/mnist.

## Author Contributions

JA wrote the paper and performed the simulations. Both JA and KR helped with developing the concepts, conceiving the experiments, and writing the paper.

### Conflict of Interest

The authors declare that the research was conducted in the absence of any commercial or financial relationships that could be construed as a potential conflict of interest.

## Footnotes

^1^Other input encodings that use time-encoding such as rank-order may provide energy efficiency improvements but at the moment provide no obvious benefit in addressing catastrophic forgetting and are thus beyond the scope of this work.

^2^Non-instantaneous potentiation kernels, such as the alpha response, are beyond the scope of this work due to the added difficulty to event-driven simulation.
