# Supplementary material for: Controlled Forgetting: Targeted Stimulation and Dopaminergic Plasticity Modulation for Unsupervised Lifelong Learning in Spiking Neural Networks
Source: Front Neurosci. 2020 Jan 28;14:7. doi: 10.3389/fnins.2020.00007 (PMC6999159; doi:10.3389/fnins.2020.00007)
Supplement: Supplementary file 1 [file Data_Sheet_1.PDF]

Supplementary Material

1 ALTERNATIVE DERIVATION OF EXPECTED VALUE OF PRE-FIRING MEMBRANE POTENTIAL

Let $V_j(t)$ be the random variable representing the potential of neuron j at time t. First, we consider the effect of the most recent incoming spike k which is received from input i at time t_{ik}. If at the time immediately before the spike was received, t_{ik}^-, the voltage of neuron j over the resting potential v_{rest} is given by $v_j(t_{ik}^-)$, then, assuming neuron j has not since fired, the potential of neuron j at time t is given by:

$$v_j(t) = \big(v_j(t_{ik}^-) + w_{ij}u(t - t_{ik})\big)e^{-(t-t_{ik})/\tau_{mem}} + v_{rest} \tag{S1}$$

where $u(t)$ is the unit step function. If we assume that the input spike has already occurred, i.e. $t > t_{ik}$, then the unit step function may be removed. Provided that the decay rate τ_{mem} remains constant, we see that the value of the potential immediately preceding the spike, $v_j(t_{ik}^-)$, and the weighted potential increase induced by the spike, w_{ij}, may be linearly separated. Without loss of generality, this linear separability may be extended to the residual potential increases induced by all spikes received by a neuron since its last firing event and refractory period, given by:

$$v_j(t) = v_{rest} + \sum_i \sum_k w_{ij}e^{-(t-t_{ik})/\tau_{mem}} \tag{S2}$$

As discussed in the text, we assume that $v_{reset} = v_{rest}$. In cases where this does not hold, the reset voltage is also linearly separable, and its residual effect may simply be added as $+(v_{reset} - v_{rest})e^{-(t-t_{last\_ref})/\tau_{mem}}$ where t_{last_ref} is the time since the last refractory period.

Since $t_{|ik|} = t - t_{ik}$ follows the distribution of gamma random variable $T_{|ik|} \sim gamma(\alpha = k; \beta = \lambda_i)$, let

$$V_{|ik|} = u(T_{|ik|}) = e^{-T_{|ik|}/\tau_{mem}} \tag{S3}$$

be the random variable representing the unweighted portion of the potential increase from the kth most recent spike from i. This allows us to rewrite (S2) as a random variable:

$$V_j = v_{rest} + \sum_i \sum_k w_{ij}V_{|ik|} \tag{S4}$$

Because $T_{|ik|}$ is a gamma random variable, we know its CDF is

$$F_{T_{|ik|}}(t) = \frac{\gamma(k, \lambda_i t)}{\Gamma(k)} \tag{S5}$$

where $\Gamma(s)$ and $\gamma(s, x)$ are the gamma function and the lower incomplete gamma function, respectively.

We can use (S5) and invert (S3) as (S6) to solve for the transformation from $T_{|ik|}$ to $V_{|ik|}$ in (S7).

$$t_1 = u^{-1}(v) = -\tau_{mem} \ln v \tag{S6}$$

$$F_{V_{|ik|}}(v) = 1 - F_{T_{|ik|}}(t_1) = \frac{\Gamma(k, -\lambda_i \tau_{mem} \ln v)}{\Gamma(k)} \tag{S7}$$

where $\Gamma(s, x)$ is the upper incomplete gamma function.

We take the derivative of the CDF in (S7) to give us the following PDF for $V_{|ik|}$:

$$f_{V_{|ik|}}(v) = \frac{(\lambda_i \tau_{mem})^k v^{\lambda_i \tau_{mem} - 1} (-\ln v)^{k-1}}{(k-1)!} \tag{S8}$$

We use the pdf of $V_{|ik|}$ to calculate its expected value in (S9), and since expectation is a linear operator, we solve for the expectation of V_j in (S10) from (S4) and (S9).

$$E(V_{|ik|}) = \int_0^1 v f_{V_{|ik|}}(v) dv = \frac{(\lambda_i \tau_{mem})^k}{(1 + \lambda_i \tau_{mem})^k} \tag{S9}$$

$$E(V_j) = v_{rest} + \sum_i \sum_k w_{ij} \frac{(\lambda_i \tau_{mem})^k}{(1 + \lambda_i \tau_{mem})^k} \tag{S10}$$

If we take the sum of spikes to infinity to get the steady state, which is a reasonable approximation since only the most recent spikes have a significant impact on the potential, then the inner sum converges to:

$$\begin{aligned} E(V_j) &\approx v_{rest} + \sum_i w_{ij} \lambda_i \tau_{mem} \\ &\approx v_{rest} + \tau_{mem} (\vec{w_j} \bullet \vec{\lambda}) \end{aligned} \tag{S11}$$

which includes a simple scaled dot product of neuron j's weight vector w_j and the input rate vector λ, as you would find in a non-spiking neuron. The appropriateness of this approximation is strengthened by the fact that in paper, the equation is used not to determine the precise spiking rate of an individual neuron but rather to compare relative spiking rates between competing neurons.

2 ADDITIONAL LIFELONG LEARNING RESULTS OVER TIME

Figure S1 shows the combined, across-task accuracy over time for the CFNs of all sizes. Figure S2 shows the per-task false positives throughout the training process as new tasks are added for networks of size 6400, showing that in the CFN case, the errors that do occur are not concentrated on one class.

3 CFN DOPAMINERGIC SPIKING ACTIVITY DURING TRAINING

Figure S3 shows the dopaminergic spiking activity during the training process using CFNs on the disjoint MNIST dataset. Note how dopaminergic activity suddenly increases each time a task change occurs and novel data is presented, followed by a gradual decrease in dopaminergic activity as the CFN learns the new

task. These activity statistics validate the assumption that low spiking activity in the non-dopaminergic neurons (which triggers the dopaminergic neuron) is a meaningful measure of novelty.

This expected variation in dopaminergic activity is most pronounced in the larger networks that have capacity to learn many different digit representations. For example, in the CFN with 6400 neurons the dopaminergic activity reaches near-zero levels by the end of learning each task. In the very small networks, the decay of dopaminergic activity is slower because a smaller network capacity means that even within a class there are more representations than capacity and thus a subset of less common but previously-seen representations continue to be viewed as “novel” since the network did not have capacity to ever permanently learn them.

Also note how different classes require different dopaminergic activity than others. For example with digit ‘1’ there is very little required dopaminergic activity because there are fewer significantly different representations of that digit within the class. Once the CFN has learned these few representations, the remaining presentations are easily recognized, reducing the need for dopaminergic assistance.

4 CFN NEURON ACTIVITY DURING TESTING

Making a shallow network wider has only marginal improvements in accuracy. However, these neurons are still used, even if infrequently. Figure S4 shows the spiking activity distribution across neurons for the CFNs during testing of all digits at the end of learning in the disjoint scenario. For each size, all or almost all of the neurons experience firing activity during testing, with only 0.7% of the neurons in the large 6400 network having zero spikes. However, the “win” counts–the number of samples that each neuron was the highest-spiking neuron–indicate that only just over half of the 6400 neurons in the large CFN were ever a decisive neuron during testing.

There are indeed a few overfit representations, as indicated by the distorted tails in the spiking distributions. These are the result of rapidly learned novel data in the last class (digit ‘9’) that didn’t experience generalization but were not subsequently overwritten because there was no following task. However, since most of the non-decisive neurons do experience some spiking activity, we can assume that a majority of them are likely not overfit representations and may therefore be useful for a different testing set selection. Additionally, this additional space may be viewed as a “scratchpad” or working memory for temporarily storing novel inputs until it is determined if they will be kept.

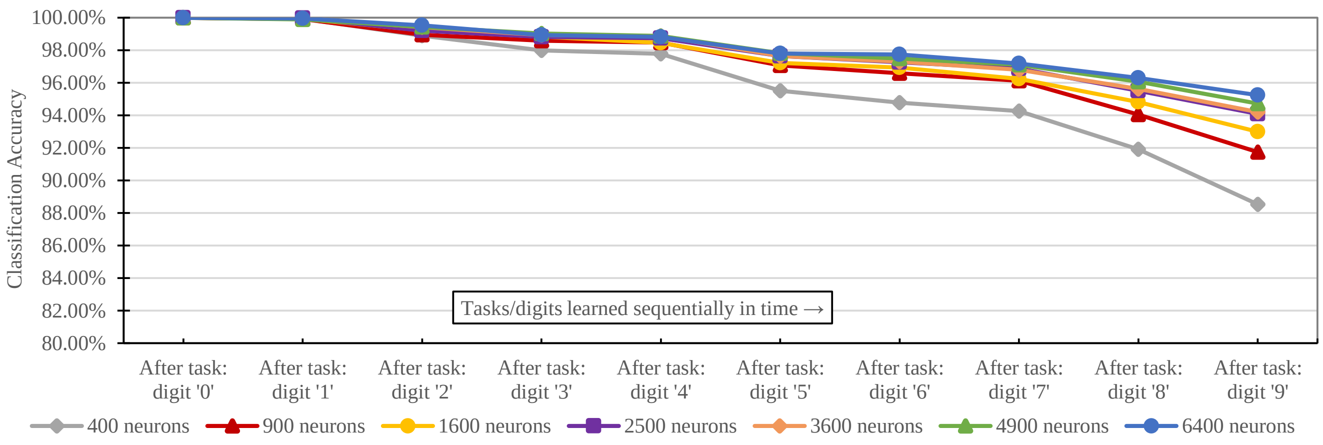

Figure S1. CFN classification accuracy over time as the number of tasks increases. Accuracy shown at each stage of the learning process (i.e. after each new task/digit) for CFNs of each size. (Five seeds.)

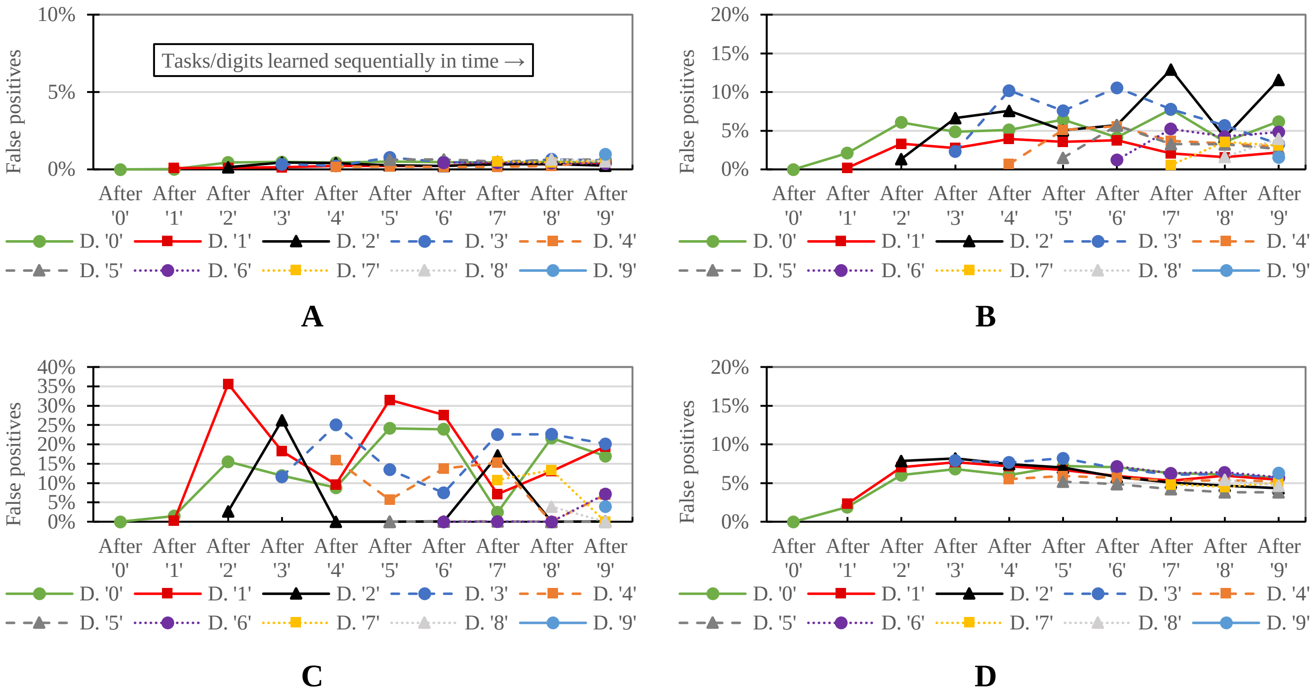

Figure S2. Per task/digit false positives as new tasks/digits are added over time for the following networks of size 6400 neurons: **(A)** the proposed CFN, **(B)** a no dopamine SNN with homeostasis **(C)** a no dopamine SNN without homeostasis, and **(D)** an SNN with random weights. (Note: vertical scales differ between charts because of the wide variation; grid lines remain consistent at 5% intervals.)

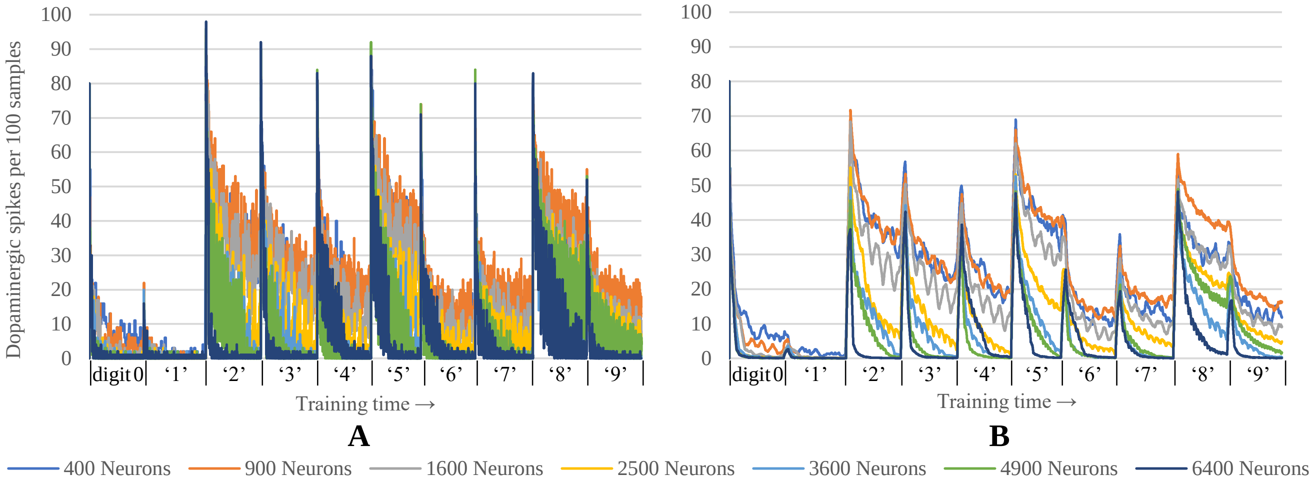

Figure S3. Dopaminergic spiking activity during training on the disjoint MNIST with CFNs. Values represent **(A)** dopaminergic spikes per 100 samples and **(B)** the same data smoothed using a running average over 100 bins.

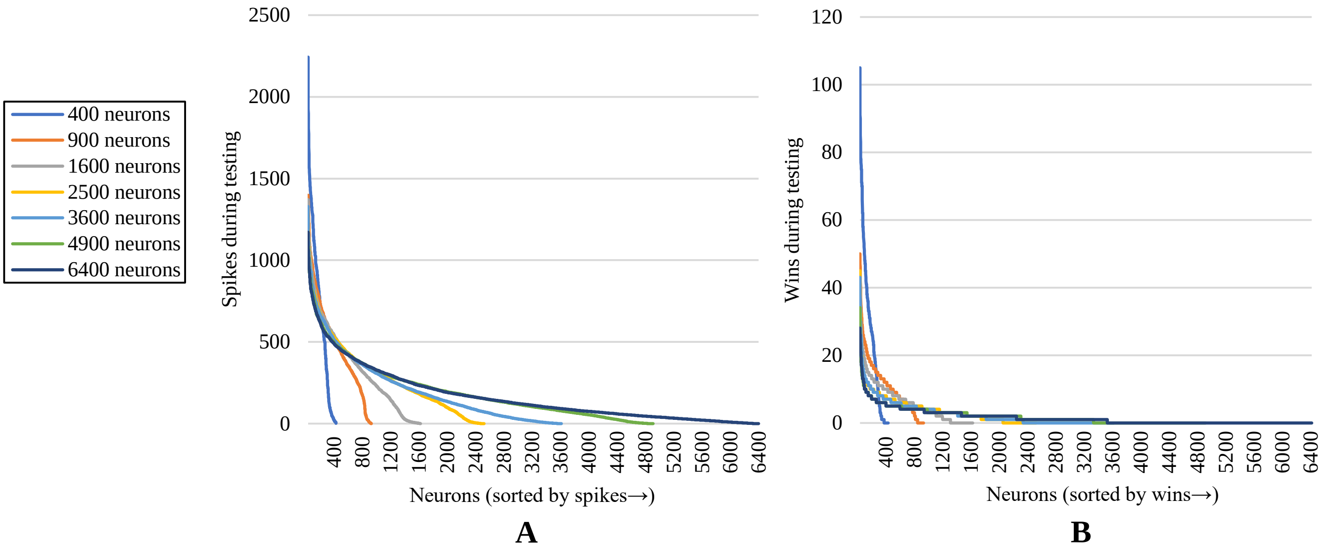

Figure S4. Activity distribution of neurons in the CFNs during testing on the disjoint MNIST. Values represent **(A)** spikes for each neuron during testing and **(B)** wins (number of samples for which each neuron was the highest spiking neuron).
